# RECQL4 Inhibits Radiation‐Induced Tumor Immune Awakening via Suppressing the cGAS‐STING Pathway in Hepatocellular Carcinoma

**DOI:** 10.1002/advs.202308009

**Published:** 2024-02-21

**Authors:** Weifeng Hong, Yang Zhang, Siwei Wang, Zongjuan Li, Danxue Zheng, Shujung Hsu, Jian Zhou, Jia Fan, Zhesheng Chen, Xiaojun Xia, Zhaochong Zeng, Qiang Gao, Min Yu, Shisuo Du

**Affiliations:** ^1^ Department of Radiation Oncology Cancer Center Zhongshan Hospital Fudan University Shanghai 200000 China; ^2^ Department of Medical Oncology Shanghai Pulmonary Hospital Tongji University School of Medicine Shanghai 200000 China; ^3^ Department of Liver Surgery and Transplantation and Key Laboratory of Carcinogenesis and Cancer Invasion (Ministry of Education) Liver Cancer Institute Zhongshan Hospital Fudan University Shanghai 200000 China; ^4^ Department of Pharmaceutical Sciences College of Pharmacy and Health Sciences; Institute for Biotechnology St. John's University Queens New York NY10003 USA; ^5^ State Key Laboratory of Oncology in South China Collaborative Innovation Center for Cancer Medicine Sun Yat‐sen University Cancer Center Guangzhou 510060 China; ^6^ Department of Pancreas Center Guangdong Provincial People's Hospital Guangdong Academy of Medical Sciences Southern Medical University Guangzhou Guangdong 510000 China

**Keywords:** cGAS‐STING pathway, DNA repair, hepatocellular carcinoma, RecQ‐Like Helicase 4, tumor microenvironment

## Abstract

Many patients with hepatocellular carcinoma (HCC) respond poorly to radiotherapy despite remarkable advances in treatment. A deeper insight into the mechanism of sensitivity of HCC to this therapy is urgently required. It is demonstrated that RECQL4 is upregulated in the malignant cells of patients with HCC. Elevated RECQL4 levels reduce the sensitivity of HCC to radiotherapy by repairing radiation‐induced double‐stranded DNA (dsDNA) fragments. Mechanistically, the inhibitory effect of RECQL4 on radiotherapy is due to the reduced recruitment of dendritic cells and CD8^+^ T cells in the tumor microenvironment (TME). RECQL4 disrupts the radiation‐induced transformation of the TME into a tumoricidal niche by inhibiting the cGAS‐STING pathway in dendritic cells. Knocking out STING in dendritic cells can block the impact of RECQL4 on HCC radiosensitivity. Notably, high RECQL4 expressions in HCC is significantly associated with poor prognosis in multiple independent cohorts. In conclusion, this study highlights how HCC‐derived RECQL4 disrupts cGAS‐STING pathway activation in dendritic cells through DNA repair, thus reducing the radiosensitivity of HCC. These findings provide new perspectives on the clinical treatment of HCC.

## Graphical abstract

Most patients with hepatocellular carcinoma respond poorly to radiotherapy. A deeper insight into the mechanisms of sensitivity to radiotherapy is urgently needed. Herein, a previously unexplored mechanism is revealed linking DNA repair, the cGAS‐STING pathway, the tumor microenvironment, and radiotherapy sensitivity, and offer an attractive novel target for HCC radiotherapy, which may be beneficial to patients with hepatocellular carcinoma.

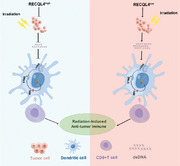

## 1 Introduction

Hepatocellular carcinoma (HCC) accounts for 80–90% of primary malignancies in the liver, and is the fifth leading cause of cancer‐related deaths worldwide.^[^ ^1^ ^]^ Despite efforts to develop novel drug targets and treatments, surgery remains the best choice.^[^ ^2^, ^3^ ^]^ Unfortunately, most patients do not meet the surgical selection criteria.^[^ ^4^ ^]^ Therefore, radiotherapy (RT), particularly combination therapy, may be the next most promising treatment method due to technological advancements.^[^ ^5^, ^6^ ^]^ Notably, a previous prospective clinical study (NCT03857815) demonstrated that stereotactic radiation can improve the efficacy of PD‐1 inhibitors in HCC. However, the exact regulatory mechanisms underlying the radiosensitivity of HCC are poorly understood.

DNA double‐strand breaks (DSBs) caused by ionizing radiation (IR) trigger cellular protective pathways, leading to the arrest of specific immune checkpoints in the cell cycle that allow for DNA damage repair (DDR).^[^ ^7^, ^8^ ^]^ In this scenario, tumor cells typically resist the cell growth‐inhibitory or cytotoxic effects of RT, resulting in a state of addiction to non‐oncogenes.^[^ ^9^ ^]^ DNA damage caused by RT also promotes tumor antigenicity and enhances adjuvanticity by increasing mutability and genomic instability.^[^ ^10^ ^]^ Additionally, RT can induce a low tumor mutation burden to produce tumor‐specific neoantigens with immunogenicity, thereby inducing immune awakening.^[^ ^11^ ^]^ Furthermore, low‐dose RT has the potential to synergize with immunotherapy, enhancing the immune response by reshaping the tumor microenvironment (TME) even in the absence of immune cell infiltration, leading to effective tumor control.^[^ ^12^ ^]^ In light of this complicated tumor‐driven alteration, there is an urgent need to discover novel cross‐mechanisms that can affect the sensitivity of HCC and the TME to RT.

The cytoplasmic DNA sensing cyclic GMP‐AMP synthase (cGAS) – stimulator of interferon genes (STING) pathway has been reported to link RT and tumor immunity to attack the “Achilles’ heel” of tumors. When cGAS binds to DNA in the cytoplasm, the second messenger molecule, cyclic GMP‐AMP (cGAMP), is synthesized, which further binds and activates the adaptor STING and triggers a signal cascade response to induce type I interferons (IFNs) and other immune molecules.^[^ ^13^, ^14^ ^]^ In addition, cGAS activation is closely related to DNA damage‐induced genome instability, which leads to the formation of micronuclei, known as the cGAS‐STING activation platform.^[^ ^15^ ^]^ Recently, ISG15 was shown to promote replication fork restart, leading to increased replication stress and chromosomal instability, affecting tumor immunogenicity.^[^ ^16^ ^]^ Similarly, certain homologous recombination (HR) or base‐excision repair defects have been linked to PD‐L1 induction following cGAS–STING activation.^[^ ^17^ ^]^ Interestingly, a previous study confirmed that double‐stranded DNA (dsDNA) released from HCC cells after RT can trigger the cGAS‐STING pathway and upregulate PD‐L1 expression.^[^ ^18^ ^]^ Therefore, it is hypothesized that the efficacy of HCC treatment is affected by a crucial DNA damage repair target that functions as a link between RT and the cGAS‐STING pathway in immune cells.

RecQ‐Like Helicase 4 (RECQL4) is one of the five RecQ helicases present in mammalian cells, and its deficiency causes Rothmund‐Thomson Syndrome (RTS). RECQL4 promotes two major DSB repair pathways: non‐homologous end joining (NHEJ) and HR.^[^ ^19^ ^]^ The RTS and other cells lacking RECQL4 are sensitive to IR, and RECQL4 rapidly accumulates in DSBs, suggesting its involvement in DSB repair.^[^ ^20^ ^]^ RECQL4 also directly interacts with the ubiquitin E3 ligase RNF8 and participates in DSB repair pathways mediated by both NHEJ and HR. Many RNA polymerase II subunits have been identified as interacting proteins with RECQL4, indicating their involvement in transcription.^[^ ^21^ ^]^ However, the function and mechanism of RECQL4 as well as TME in HCC have not yet been investigated.

In this study, RECQL4 was identified as a key DDR marker for malignant HCC cells at the single‐cell level using single‐cell RNA sequencing (scRNA‐seq). Using various genetically engineered mouse models, we found that tumor‐derived RECQL4 inhibited cGAS‐STING pathway activation in dendritic cells and anti‐tumor immune reorganization after RT, thereby reducing the sensitivity to HCC treatment. Finally, our analysis of multiple large datasets comprising of 1216 HCC patients, including the Fudan University Zhongshan Hospital (FDUZS) cohort, revealed that RECQL4 is highly expressed in HCC cells and is significantly correlated with poor prognosis. Overall, our findings pioneered a previously unexplored mechanism of post‐RT immunomodulation, which indicates a therapeutic strategy for disrupting DDR dependencies in radiation sensitization and provides potential predictive markers for monitoring HCC efficacy.

## 2 Results

### 2.1 DNA Repair Marker Capabilities of RECQL4 for Patients with HCC

**Figure 1:**
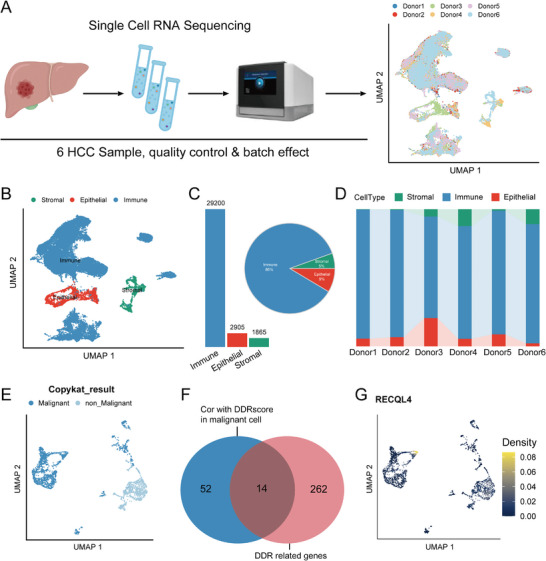
Deep dissection of HCC scRNA‐seq reveals RECQL4 was highly expressed in malignant cells. A) Schematic diagram of scRNA‐seq data acquisition. B) UMAP plot representing cell types. C) Bar graph and pie chart showing relative numbers and cell proportion of different cell types, with each color representing a relevant cell type. D) Relative percentage of three cell clusters in different sample sources. E) UMAP plot representing malignant and non‐malignant cells detected by CopyKAT. F) Venn plot represents the intersection between genes significantly associated with DDRscore in malignant cells and 276 DDR genes. F) Density plot showing the expression distribution of RECQL4 in epithelial cells.

A total of 43786 cells were obtained from the six HCC samples, which were subjected to scRNA‐seq analysis, as illustrated in **Figure** **1**A (left panel). After quality control and screening procedures, 33970 cells were retained and used to draw up gene expression profiles. Clustering was performed after expression normalization and batch effect removal. Using UMAP intersample variation, pre‐existing biological differences between samples were shown (Figure 1A, right panel). To gain a closer view of the HCC tumor microenvironment, all the cells were merged and classified into stromal, immune, and epithelial cells based on the identified feature genes (Figure 1B). A total of 1865 stromal, 29 200 immune, and 2905 epithelial cells were assigned to the clusters for subsequent analysis (Figure 1C). Immune and epithelial cells were largely dominant (Figure 1C). The proportions of the three cell types across the samples (Figure 1D). A total of 1965 malignant and 940 non‐malignant cells in the epithelial cell population were stratified using the CopyKAT algorithm (Figure 1E; Figure S1A, Supporting Information).

Since DDR pathways play crucial roles in maintaining genomic stability, defects within DDR pathways lead to the accumulation of damaged DNA, resulting in tumorigenesis, disease progression, and alterations in therapy‐associated responses.^[^ ^22^ ^]^ The DDR status (DDR score) of malignant cells were evaluated at a single‐cell resolution based on the 276 DDR‐related genes detected (Figure S1B, Supporting Information). Spearman's analysis was performed to identify mRNA transcripts and exhibited a significant correlation with DDR scores in malignant cells. The analysis further revealed that 14 of these genes were DDR‐related (Figure 1F). Interestingly, the results revealed an exclusive expression of RECQL4 in malignant cells (Figure 1G; Figure S1C–O, Supporting Information). Since RECQL4 is an important hallmark molecule for stratifying HCC patients, it may be involved in T cell‐mediated cancer cell sensing and killing,^[^ ^23^, ^24^ ^]^ we propose that RECQL4 might serve as a potential target for DNA damage repair in HCC in a designated subpopulation of HCC patients.

### 2.2 Radiosensitivity of HCC in Relation to RECQL4

The functional role of RECQL4 in HCC was investigated by comparing its expression in normal human liver cell lines (WRL‐68 and L02) and HCC cell lines (MHCC97H, Hep3B, Huh7, MHCC97L, HCCLM3, and HepG2) using qPCR and western blotting (WB). Results from the qPCR and WB showed that RECQL4 was upregulated at both the mRNA and protein levels in almost all the HCC cell lines assessed, albeit at different magnitudes (Figure S2A,B, Supporting Information). Concordantly, RECQL4 was highly expressed in HCCLM3 cells, but minimally expressed in MHCC97H cells. Therefore, the MHCC97H and HCCLM3 cell lines were selected for subsequent functional gain‐and‐loss assays. RECQL4 was stably overexpressed via plasmid transfection in MHCC97H, while effectively downregulated using three different shRNAs in the HCCLM3. Overexpression and stable knockdown of RECQL4 in MHCC97H and HCCLM3 cells were verified by qPCR. (Figure S2C,D, Supporting Information). As shRECQL4‐1 had the most significant inhibitory effect, it was used in subsequent experiments.

**Figure 2:**
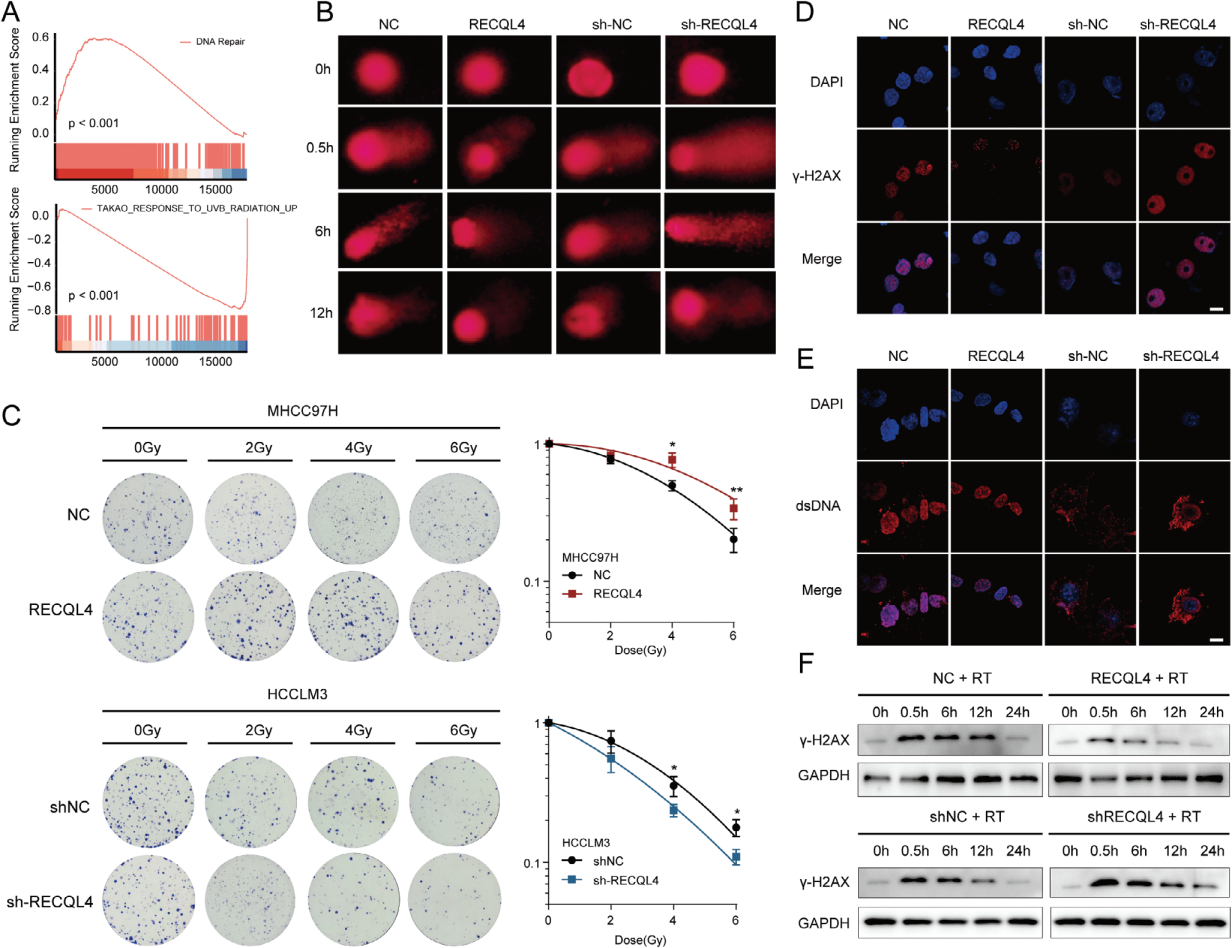
RECQL4 accelerates DNA damage repair in HCC after radiotherapy in vitro. A) GSEA of gene expression data in FDUZS cohort shows RECQL4 expression were enriched in DNA repair pathway and radiation response pathways. B) Representative images of neutral comet assay at 0, 0.5, 6, and 12 h after IR. C) Clonogenic assays and survival fraction curves of MHCC97H and HCCLM3 cells stably transfected with RECQL4 or empty vector after exposure to the indicated IR dose. D,E) Immunofluorescence staining revealed the cellular location of γ‐H2AX (D) and dsDNA (E) at 10 h after exposure to 6 Gy IR. F) Western blotting detects γ‐H2AX at different time points after RT in MHCC97H cells treated with control or RECQLE‐overexpression, or HCCLM3 cells treated with shNC or shRECQL4. Scale bar, 10 µm. Data are presented as mean ± sd.

The Gene Set Enrichment Analysis (GSEA) of the FDUZS cohort revealed that RECQL4 expression was remarkably enriched in DNA repair and radiation response pathways (**Figure** **2**A). DNA damage is the main form of cell death caused by RT, and inhibition of repair after DNA damage can increase the cell‐killing effect induced by IR. The effect of RECQL4 on IR‐induced DNA damage in HCC cells was investigated. Comet tail motion, which reflects IR‐induced physical killing, showed that RECQL4 overexpression effectively inhibited IR‐induced DNA damage compared to that in the control group, whereas RECQL4 knockdown exhibited the opposite trend (Figure 2B). Furthermore, a clonogenic assay was performed to assess cell function after exposure to IR, the results of this showed that RECQL4 overexpression improved colony formation and proliferation of MHCC97H cells after IR. Conversely, RECQL4 knockdown in HCCLM3 cells severely impeded cell survival and proliferation following IR‐induced DNA damage caused by IR (Figure 2C). Immunofluorescence in situ detection of γ‐H2AX foci also confirmed the association of RECQL4‐overexpression to cause shortened duration of γ‐H2AX foci, a trend that can be reversed via RECQL4‐knockdown after IR exposure (Figure 2D). Immunofluorescence analysis of dsDNA showed that RECQL4‐overexpression decreased dsDNA production following RT, while RECQL4‐knockdown increased dsDNA levels (Figure 2E). The result of WB demonstrated that the levels of γ‐H2AX decreased faster with time in the RECQL4‐overexpression group than in the control group after exposure to IR, while RECQL4‐knockdown increased IR‐induced γ‐H2AX expression in HCC cells (Figure 2F).

### 2.3 Radiation Sensitivity of TME in the Presence of RECQL4 Expression

**Figure 3:**
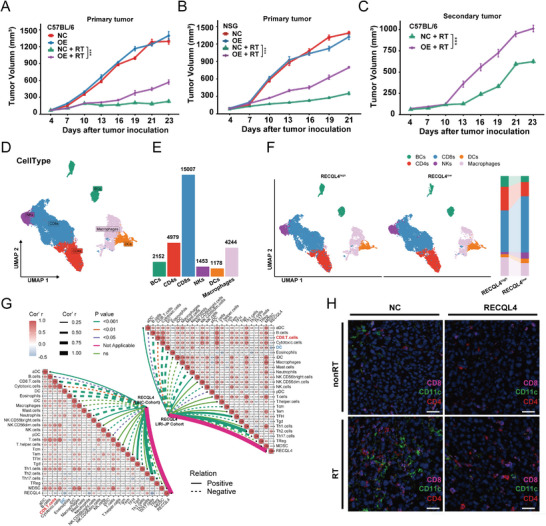
RECQL4 regulates HCC radiosensitivity and induces a suppressive TME. A,B) Growth curves of primary tumors in C57BL/6 mics and NSG mice (*n* = 6). C) Growth curves of secondary tumors in each group (*n* = 6). D) UMAP plot performs dimensionality reduction and clustering of immune cells from scRNA‐seq. E) Bar graph represents the number of various immune cell types. F) ScRNA‐seq analysis of immune cells in HCC samples with high or low RECQL4 expression (left panel). Proportional graph of immune cells grouped by high or low RECQL4 expression (right panel). G) Immune infiltration analysis from TCGA‐LIHC cohort and ICGC‐LIRI‐JP cohort shows correlation between RECQL4 and DCs/CD8+T cells. H) Representative images of multi‐color immunofluorescence analysis of mouse HCC tissue labeled with CD4 (red), CD11c (green), and CD8 (pink). Data are presented as mean ± sem. *P*<0.05, ** *P*<0.01, *** *P*<0.001, **** *P*<0.0001.

To further elucidate the relationship between RECQL4 and radiation sensitivity in HCC, we established subcutaneous xenografts by injecting overexpressed H22 cells into C57BL/6 mice and subsequently administering fractionated radiation (Figure S3A, Supporting Information). A notable hindrance in the regression of HCC after RT upon the induction of RECQL4 expression in immunocompetent C57BL/6 mice was observed (**Figure** **3**A), the degree of this impact differed from that observed in immunodeficient NSG mice (Figure 3B), suggesting that the immune system may be involved in RECQL4 function. Previous studies have demonstrated that RT can activate the immune system against cancer cells, leading to regression of distant tumors with the same antigenic effect, a phenomenon known as the “abscopal effects”.^[^ ^25^ ^]^ Due to these findings, an “abscopal effect” model was considered to investigate the impact of the intact immune system on RECQL4‐mediated HCC radiotherapy efficacy (Figure S3B, Supporting Information). Interestingly, the same fractionated dose also induced secondary tumor growth in RECQL4‐overexpression mice as compared to that in the control group (Figure 3C; Figure S3C, Supporting Information).

To gain a deeper understanding of the relationship between RECQL4 and the immune system, we used the scRNA‐seq data and annotated 29200 immune cells (Figure 3D; Figure S4, Supporting Information). There were 2152 B cells, 4979 CD4+ T cells, 15007 CD8^+^T cells, 1453 natural killer (NKs) cells, 1178 dendritic cells (DCs), and 4244 macrophages (Figure 3E). We then dissected these immune cells based on their epithelial RECQL4 expression levels across patients (namely RECQL4^high^ and RECQL4^low^ groups). Intriguingly, compared with the RECQL4^low^ group, the proportions of CD8^+^T cells, NKs cells, and DCs decreased significantly in the RECQL4^high^ group, whereas the proportions of B cells, CD4^+^T cells, and macrophages increased significantly (Figure 3F). To validate this, we investigated the relationship between RECQL4 expression and immune infiltration in HCC using external TCGA‐LIHC and ICGC‐LIRI cohorts. Immune cell deconvolution showed that RECQL4 expression in HCC correlated with low CD8^+^ T cell infiltration (TCGA‐LIHC: r = −0.23; ICGC‐LIRI: r = −0.24) and depleted DCs (TCGA‐LIHC: r = −0.41; ICGC‐LIRI: r = −0.41) (Figure 3G). To test the DNA damage‐associated response, we focused on the negative feedback upon RECQL4 expression via multi‐plex immunofluorescence. Mice inoculated with HCC cells with RECQL4 overexpression demonstrated a significant reduction in the number of DCs (CD11c^+^), CD8^+^T (CD8^+^) and CD4^+^T (CD4^+^) cells upon irradiation (Figure 3H). Collectively, these findings imply a probable role for RECQL4 in discharging DCs and CD8^+^ T cells following DNA damage.

**Figure 4:**
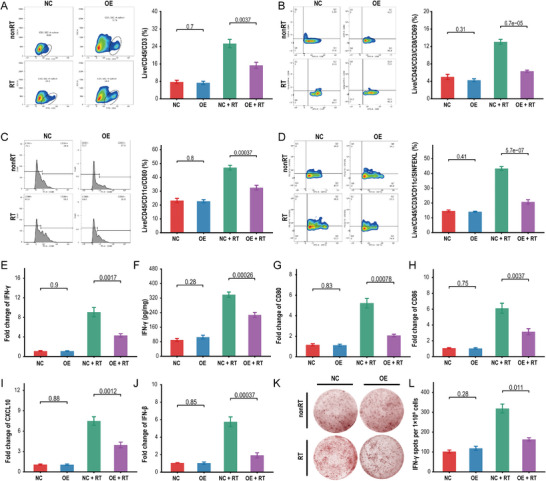
RECQL4 impairs radiation‐induced recruitment of DCs and CD8+ T cells. A,B) Flow cytometric analysis of tumor infiltrating CD3+ T cell population (Live/CD45+/CD3+ cells) (A) and CD8+ T cell population (Live/CD45/CD3/CD8/CD69) B) in WT mouse tumor tissue. C,D) Flow cytometric analysis of antigen‐specific dendritic cells (CD45+/CD11c+/SIINFEKL+ cells/CD80) in draining lymph nodes of WT mice. E) qRT‐PCR quantification of IFN‐γ expression in tumor tissue. F) ELISA measurement of IFN‐γ concentration in tumor tissue, represented as pg/10 mg tumor tissue. G–J) qRT‐PCR quantification of CD80 (G), CD86 (H), CXCL10 (I), and IFN‐β (J) expression in tumor‐draining lymph node tissue. K,L) Purified CD11c+ DCs were co‐cultured with initial CD8+ T cells, and IFN‐γ secretion was detected by ELISPOT assay. The representative data shown are from six mice per group. Data are presented as mean ± SEM.

Flow cytometry (FACS) was used to profile RECQL4‐mediated immune modulation in a mouse model. Decreased levels of CD8^+^ T cells (CD45^+^/CD3^+^/CD8^+^ cells) and antigen‐specific DCs (CD45^+^/CD11c^+^/SIINFEKL^+^ cells) in tumors/tumor‐draining lymph nodes (TDLN) in mice with subcutaneous graft HCC tumor carrying RECQL4 following irradiation‐induced DNA damage (**Figure** **4**A–D). CD8^+^ T cells were inhibited by RECQL4 overexpression in tumors (*P*<0.05) (Figure 4A,B) and DCs from TDLN tissues were also suppressed (*P*<0.05; Figure 4C,D). Additionally, overexpression of RECQL4 also suppressed the mRNA and protein expression of interferon‐γ in RT‐treated tumor tissue (Figure 4E,F). Concordantly, mRNA levels of CD80, CD86, CXCL10, and interferon‐β in TDLN also showed similar trends (Figure 4G–J). We further confirmed this using the ELISPOT assay, whereby RECQL4 overexpression significantly inhibited the production of IFN‐γ from CD8^+^ T cells induced by RT (Figure 4K–L). Taken together, our data indicate that RECQL4 reduces the migration and recruitment of DCs induced by RT, thereby diminishing the activation of CD8^+^ T cells by suppressing interferon‐γ production, facilitating the oncogenic transformation of the HCC TME.

### 2.4 RECQL4‐Mediated Immuno‐Suppression in Relation to the cGAS‐STING Pathway

**Figure 5:**
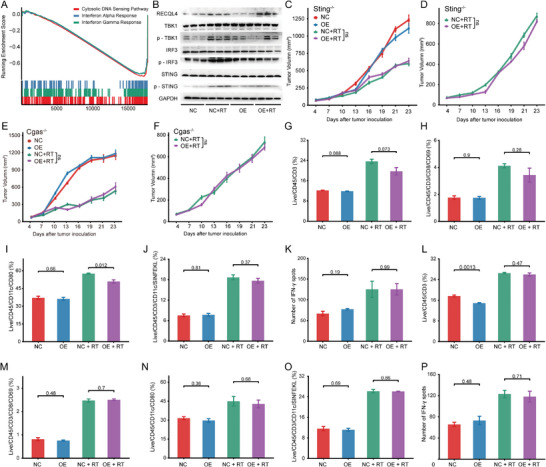
Immune suppression mediated by RECQL4 in liver cancer radiotherapy is dependent on disrupted cGAS‐STING pathway. A) GSEA of gene expression data in FDUZS cohort shows inhibition of Cytosolic DNA Sensing Pathway, Interferon Alpha Response, and Interferon Gamma Response in RECQL4 high‐expressing group. B) Western blotting analysis of RECQL4, TBK1, p‐TBK1, IRF3, p‐IRF3, STING, and p‐STING. C,D) Growth curves of primary tumors (C) and secondary tumors (D) in STING‐mutation mice in different treatment groups. E,F) Flow cytometric analysis of tumor‐infiltrating CD3+ T cell population (Live/CD45+/CD3+ cells) and CD8+ T cell population (Live/CD45/CD3/CD8/CD69) in STING‐mutation mouse tumor tissue. G,H) Flow cytometric analysis of antigen‐specific dendritic cells (CD45+/CD11c+/SIINFEKL+ cells/CD80) in draining lymph nodes of STING‐mutation mice. I) Purified CD11c+ DCs from STING‐mutation mice were co‐cultured with initial CD8+ T cells, and IFN‐γ secretion was detected by ELISPOT assay. J,K) Growth curves of primary tumors (J) and secondary tumors (K) in cGAS‐KO mice in different treatment groups. L,M) Flow cytometric analysis of tumor‐infiltrating CD3+ T cell population and CD8+ T cell population in cGAS‐KO mouse tumor tissue. N,O) Flow cytometric analysis of antigen‐specific dendritic cells in cGAS‐KO mouse tumor tissue. P) ELISPOT assay detected IFN‐γ secretion in cGAS‐KO mice.

To elucidate the downstream signaling of RECQL4, GSEA was performed in the FDUZS cohort. Notably, HCC specimens with high RECQL4 expression substantially affected the “cytosolic DNA‐sensing pathway” compared with those with low RECQL4 expressions (**Figure** **5**A). In addition, overexpression of RECQL4 also resulted in a significant inhibition of Interferon‐α/Interferon‐γ response. The cGAS‐STING pathway (a crucial sensor of cytosolic DNA) triggers the production of IFN‐I and other inflammatory cytokines following exposure to endogenous pathogen DNA.^[^ ^26^ ^]^ We propose that cGAS‐STING signaling might be involved in this process. As shown by WB, a reduction in the phosphorylation of the pathway activation of the total and phosphorylated STING, TBK1, and IRF3 genes were observed in post‐irradiated HCC overexpressing RECQL4 (Figure 5B).

The involvement of the cGAS‐STING pathway in the RECQL4‐mediated tumor‐promoting immune blockade was tested in vivo. There was no significant difference observed for Tmem173gt and Mb21d1^−⁄−^ knockout models when compared to RECQL4‐overexpression with empty controls upon RT treatment, despite the fact that irradiation induced tumor regression was observed in both conditions (Figure 5C–F). Since the aforementioned results demonstrated the function of RECQL4‐in limiting the recruitment and activation of DCs and CD8^+^ T cells under radiation‐sensitizing conditions (Figure 3H–J and Figure 4), we explored the role of cGAS‐STING in regulating RECQL4‐mediated immune‐phenotypical changes. FACS was used to detect STING‐deficient and cGAS‐deficient mice to establish the role of cGAS‐STING in this process. No significant differences were observed in the proportions of CD8^+^ T cells and DCs between the RECQL4‐overexpression and control groups. However, the proportions of CD8+ T cells and DCs were significantly higher in the RT group. RECQL4‐overexpression did not significantly affect this radiation‐induced change (Figure 5G–J; Figure S5A, Supporting Information). We continued with the ELISPOT assay to assess IFN‐γ expression. CD11c^+^ cells were purified from the TDLNs of Tmem173gt mice and tested for IFN‐γ (Figure S5B, Supporting Information). As indicated in Figure 5K, the absence of STING eliminated the DCs cross‐priming of RECQL4 injury. These observations were also noted in Mb21d1^−⁄−^ mice (Figure 5L–P; Figure S5C,D, Supporting Information). Collectively, these findings suggest that RECQL4 inhibits RT‐induced DCs antigen presentation and blocks the antitumor effects of CD8^+^ T cells in a cGAS‐STING‐dependent manner.

### 2.5 RECQL4 Impact on Dendritic Cells as the Cellular Site of Dysregulated cGAS‐STING Signaling

**Figure 6:**
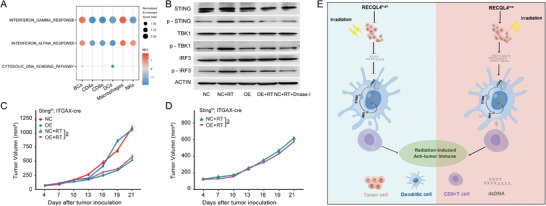
RECQL4 suppresses radiation‐induced anti‐liver cancer immune response through cGAS‐STING signaling in dendritic cells. A) Bubble plot of cell type enrichment analysis based on scRNA‐seq. B) Western blotting analysis of STING, p‐STING, TBK1, p‐TBK1, IRF3, and p‐IRF3. C,D) Growth curves of primary tumors (C) and secondary tumors (D) in STING‐cKO mice in different treatment groups. E) Proposed working model of RECQL4. RECQL4 repairs DNA damage caused by RT to HCC, reduces the production and release of dsDNA, and inhibits the cGAS‐STING signaling in dendritic cells, thereby suppressing antigen presentation and effector T cell function of dendritic cells, reducing IFN release, weakening HCC radio‐sensitivity, and blocking the anticancer effect of RT activation.

Given that RECQL4 inhibits radiation‐induced tumoricidal effects by suppressing cGAS‐STING signaling and remodeling the HCC immune microenvironment, we aimed to identify the targeting cells in which cGAS‐STING could exert its regulatory effects. An enrichment analysis comparing RECQL4^high^ and RECQL4^low^ samples on immune cell types were detected using the scRNA‐seq data. Interestingly, although IFN‐related pathways were universally present across multiple immune cell types, only DCs showed significant enrichment in the cytosolic DNA‐sensing pathway (**Figure** **6**A). To confirm whether DCs are the main cellular sites that allow RECQL4‐mediated cGAS‐STING signaling dysregulation, mouse BMDCs were cultured under different conditions. The results showed that RT‐produced dsDNA can activate the innate immune cGAS‐STING pathway, which is potentially mediated by RECQL4. Therefore DNase‐I digestion was used to remove the radiation‐generated dsDNA fragments. As shown in Figure 6B, RECQL4 overexpression along with DNase‐I treatment decreased the phosphorylation of STING, TBK1, and IRF3, primarily induced by RT in BMDCs, indicating a role for RECQL4 in attenuating the DNA‐sensing pathway in DCs. Similarly, RT‐qPCR showed significantly lower mRNA levels of CD80, CD86, CXCL10, and interferon‐I in the RECQL4 overexpressing and DNase‐I groups than in the RT group under the same experimental conditions (Figure S6A–D, Supporting Information). Interferon‐I enhances the cross‐stimulation of DCs, resulting in an acquired immune response. Therefore, the costimulatory effects on tumor‐specific T cells were studied and these were potentially induced by tumor‐derived DCs using ELISPOT assays. The results showed that RECQL4 overexpression together with DC co‐culture in the RT group significantly inhibited interferon‐γ production by CD8^+^ T cells highlighting the crosstalk between the DC and T cells to create an immune‐suppressive environment mediated via RECQL4 (Figure S6E,F, Supporting Information).

Finally, to validated the RECQL4 function in vivo, we generated conditional knockout mice for STING in DCs by fusing Sting‐cKO mice (Sting^f/f^ × ITGAX‐cre). Using this method, we established subcutaneously grafted tumors in Sting‐cKO mice and treated them with radiation (Figure 3B). It was shown that RECQL4 overexpression did not reverse the growth inhibition of primary and secondary tumors induced by RT compared to that in Sting‐cKO mice (Figure 6C,D). However, a comparison of the results of wild‐type mice, STING‐deficient mice, and Sting‐cKO mice (Figure 3C,E, Figure 5C,D, and Figure 6C,D) showed that RECQL4 significantly inhibited RT‐induced elimination of primary and secondary tumors. We confirmed that the conditional knockout of STING in DCs eliminated the inhibition of RECQL4 triggered by cGAS‐STING signaling. These findings confirm that DCs are the cellular sites of RECQL4‐damaged cGAS‐STING signaling dysregulation.

### 2.6 RECQL4 in HCC Prognosis

**Figure 7:**
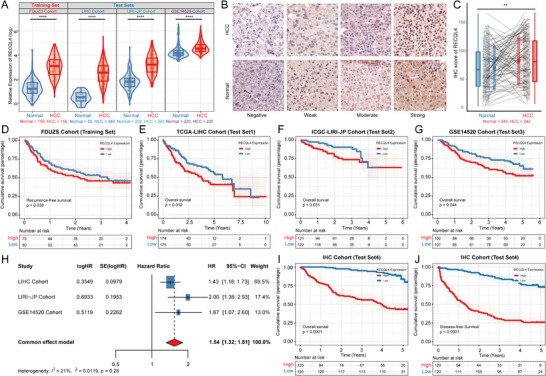
Overexpression of RECQL4 in HCC is associated with poor prognosis. A) RECQL4 expression levels in 976 cases of HCC and non‐tumor tissues from four independent cohorts (FDUZS, TCGA‐LIHC, ICGC‐LIRI‐JP, and GSE14520). B,C) Representative images and statistical analysis of RECQL4 IHC staining intensity in 240 pairs of tissues. Scale bar, 50 µm. D) Kaplan‐Meier RFS curve of HCC tissues with high and low RECQL4 levels in FDUZS cohort (*n* = 159). E–G) Kaplan‐Meier OS curves of HCC tissues with high and low RECQL4 levels in three independent cohorts (TCGA‐LIHC cohort, *n* = 349; ICGC‐LIRI‐JP cohort, *n* = 243; GSE14520 cohort, *n* = 225). H) Meta‐analysis model integrating OS prognostic analysis results from three independent cohorts. I,J) Kaplan‐Meier OS and DFS curves of HCC tissues with high and low RECQL4 IHC‐score after quantifying IHC staining intensity (FDUZS cohort, *n* = 240). * *P*<0.05, ** *P*<0.01, *** *P*<0.001, **** *P*<0.0001.

To evaluate the expression of RECQL4 and its potential association with the clinical characteristics of HCC, we conducted a comprehensive next‐generation sequencing (NGS) analysis of tumor and matched adjacent non‐tumor tissues obtained from 159 patients with HCC at Fudan University Zhongshan Hospital (FDUZS cohort) as a training set and used three independent HCC datasets from public repositories for validation. A total of 817 tumors and 472 adjacent tissues were identified (TCGA‐LIHC cohort: 349 tumors and 50 adjacent tissues; ICGC‐LIRI‐JP cohort: 243 tumors and 202 adjacent tissues; GSE14520 cohort: 225 tumors and 220 adjacent tissues). The training set analysis revealed a significant upregulation of RECQL4 expression in HCC samples compared to peritumoral samples. This finding was also recapitulated across all three independent validation cohorts (*P*<0.0001, **Figure** **7**A). IHC was used to assess the protein expression of RECQL4 in 240 patients with HCC. It was confirmed that RECQL4 was upregulated in tumor tissues compared to that in adjacent non‐tumor controls (*P*<0.0001, Figure 7B,C).

We also examined the association between RECQL4 expression and HCC patient survival. Kaplan‐Meier analysis revealed a significant association between elevated RECQL4 expression and shorter relapse‐free survival RFS (*P* = 0.038) among HCC patients in the FDUZS cohort (Figure 7D). Moreover, high RECQL4 expression in the validation cohorts (TCGA‐LIHC, ICGC‐LIRI‐JP, and GSE14520) was associated with poor overall survival (OS) (*P* < 0.05) (Figure 7E–G). To eliminate potential analytical biases across different cohorts, a fixed‐effects meta‐analysis model was used to assess OS outcomes across three independent cohorts. These findings showed that elevated expressions of RECQL4 was a key indicator of adverse clinical outcomes in HCC (HR [95% CI]:1.54 [1.32–1.81], Figure 7H). Furthermore, the IHC staining intensity (IHC score) was quantified and the corresponding survival characteristics of this study's cohort was analyzed. The results showed that higher RECQL4 expressions was significantly associated with shorter OS and disease‐free survival (DFS) (Figure 7I–J). Finally, we collected tissues from 14 HCC patients who received SBRT in conjunction with immunotherapy. We utilized IHC to assess the expression of RECQL4. The results indicate that high expression of RECQL4 may suggest non‐responsiveness to the combined therapy of radiation and immunotherapy (stable disease/progressive disease, Figure S7, Supporting Information). Collectively, our results suggested that RECQL4 is a critical gene associated with poor prognosis in HCC.

## 3 Discussion

DNA damage response plays a critical role in repairing DNA damage and maintaining genome stability.^[^ ^27^, ^28^ ^]^ However, tumor cells can also escape cell death by enhancing their DNA damage repair capacity, leading to resistance to RT or chemotherapeutic drugs.^[^ ^29^, ^30^ ^]^ This “double‐edged sword” effect highlights the need to identify new key regulatory factors involved in the tumor cell DDR pathway and the modulation of RT or chemotherapy drug resistance as a potential strategy to improve cancer treatment efficacy. Although studies have extensively described the DDR characteristics of tumor cells from the perspective of scRNA‐seq and have analyzed their impact on TME remodeling,^[^ ^31^, ^32^ ^]^ the exact regulatory factors and their precise mechanisms remain unclear.

RECQL4 is an important DDR marker that promotes both NHEJ and HR.^[^ ^33^ ^]^ The HR pathway can cause HR defects that cannot repair dsDNA breaks, making tumor cells highly sensitive to platinum drugs and poly‐ADP‐ribose polymerase (PARP) inhibitors. This has become a diagnostic and therapeutic strategy for various cancers, including ovarian and pancreatic cancer.^[^ ^34^ ^]^ However, the role of RECQL4 in the radiation‐induced tumor response in HCC has not yet been reported. Our data confirmed that RECQL4 promotes DNA damage repair after liver cancer radiotherapy, mainly by repairing dsDNA. In addition, RECQL4 overexpression counteracted the therapeutic effect of RT on HCC and abscopal effects. Mechanistically, we demonstrated that HCC‐derived RECQL4 can act as a major immune controller in the TME by suppressing cGAS‐STING activation in radiation‐induced DCs and reducing the release of IFN‐I in CD8 T cells. This process strengthens the immune escape and reduces the sensitivity of HCC to RT. Multiple accurate gene knockout mice confirmed that when cGAS/STING deficiency occurs, RECQL4 overexpression eliminates RT antagonism, restoring the ability of DCs to mediate CD8^+^ T cell cross‐activation and suggesting crosstalk between tumor cell dsDNA damage repair and programmed T cell activation. This finding explains a new mechanism of tumor cell resistance to RT and suggests a novel HCC target that may aid in the clinical development of combination therapy strategies.

Its unique anatomical position in the liver exposes it to various antigens, including dietary and commensal proteins. Over time, the liver acquires the ability to regulate immune responses against harmless antigens, thereby forming an immune‐tolerant microenvironment that affects HCC development and treatment.^[^ ^35^ ^]^ In addition, whether a molecule can become a target for cancer therapy depends on two necessary features: TME‐specific overexpression and immune‐inhibitory function.^[^ ^36^ ^]^ Single‐cell sequencing landscapes and bulk sequencing analyses revealed a significant correlation between RECQL4 and the decreased recruitment of DCs and CD8^+^ T cells. This finding was validated in mice using immunofluorescence, which demonstrated that RECQL4 antagonized the communication between tumor cells and the immune system induced by RT. We tested the hypothesis that RECQL4 overexpression in mice with liver cancer receiving radiation therapy reduces tumor sensitivity to RT by antagonizing STING‐dependent elimination of cancer cells. This antagonistic effect may arise from interference between dsDNA released by tumor cells and DCs, thereby affecting DC antigen presentation and reducing the stimulation of CD8^+^ T cells. Mismatch repair‐deficient tumors induce stronger DC‐mediated cross‐activation of CD8^+^ T cells, and increased DNA sensing in tumor cells, therefore promoting adaptive immunity.^[^ ^37^ ^]^ In various treatment environments such as chemotherapy, radiotherapy, and DNA repair pathway‐targeted therapy, damaged DNA enhances T cell activation and antitumor effects by triggering Toll‐like receptors or cGAS‐STING‐dependent IFN‐I signaling.^[^ ^38^, ^39^ ^]^ Radiation‐induced activation of cGAS‐STING in DCs is essential for tumor suppression in the immune‐tolerant microenvironment of the liver. In STING‐deficient and cGAS‐deficient mice, the disappearance of CD8+ T cells increased with RT. The cGAS‐STING pathway is a key regulator of tumor CD8+ T cell infiltration and is regulated by RECQL4. There are two possible explanations for this phenomenon. First, IR can cause acute tumor‐related death; Second, in the TME, tumor DNA and cyclic GMP‐AMP are released, leading to activation of the cGAS‐STING pathway.^[^ ^40^ ^]^ However, RECQL4 reduces tumor cell damage, resulting in a reduction in dsDNA release and adaptive immune responses caused by cGAS‐STING activation in DCs. On the other hand, RECQL4 overexpression reduces the recruitment of DCs and CD8+ T cells, reducing the “soil” on which cGAS‐STING and IFN‐I are produced, leading to tumor immune evasion.

Multiple intersections appear in the response of the TME to radiation. Radiation activates inflammatory pathways, promotes DC maturation, increases T‐cell initiation, and makes tumor cells more sensitive to immune recognition. In other words, localized inflammatory responses caused by radiation can reset the interference between tumors and the immune system, induce immune stimulation signals to increase tumor antigen presentation, activate effector T cells, create an immune‐permissible TME, and enhance responsiveness to immunotherapy.^[^ ^41^ ^]^ Recent studies have shown that exhausted T cells (Tex) promote MDSC differentiation, leading to an antigen‐presenting phenotype.^[^ ^42^ ^]^ CD8^+^ Tex cells, which kill tumors, originate from precursor‐exhausted T cells (Tpex). Owing to ICB treatment, Tpex cells have a stronger ability to proliferate and differentiate into Tex cells, enhancing immune responses.^[^ ^43^, ^44^ ^]^ Therefore, we speculated that RECQL4 impairs the transformation of the radiation‐induced TME into a tumor‐killing ecological niche, which is detrimental to immune checkpoint inhibitor therapy and may lead to resistance to RT combined with immune therapy. However, this prediction requires confirmation in large‐scale clinical trials.

In summary, our data provided the first evidence of a relationship between tumor‐derived RECQL4 and cGAS/STING activation in radiation‐triggered DCs in HCC (Figure 6E). RECQL4 repairs dsDNA released by tumor cells during RT, suppresses cGAS‐STING signaling in DCs, and prevents radiation‐induced tumor immune awakening. Our study lays the foundation for a better understanding of the mechanisms underlying radiation resistance and identifies potential targets for the treatment of HCC.

## 4 Experimental Section

### Patient Recruitment and Data Collection

Postoperative tumor samples from six patients with HCC were received from the Guangdong Provincial People's Hospital, China (Approved by the Ethics Committee of Guangdong Provincial People's Hospital, China, No. KY‐Q‐2022‐379‐01). The inclusion criteria for cases are as follows: (i) Patients diagnosed with hepatocellular carcinoma (HCC) based on pathology; (ii) Classified as Stage A according to the Barcelona Clinic Liver Cancer (BCLC) staging system; (iii) Possessing comprehensive radiological information. Additionally, 6 independent cohort studies were included. (i) FDUZS Cohort 1: 159 patients with HCC had paired tumors and adjacent non‐tumor liver tissues, previously described by Gao et al..^[^ ^45^ ^]^ (ii) FDUZS Cohort 2: The tissue microarray (TMA) technique was used to prepare HCC and paired para‐cancerous tissues collected from Zhongshan Hospital Fudan University, China. Representative tumor regions were obtained from formalin‐fixed and paraffin‐embedded primary cancer tissues, and a 1.5 mm core of each tumor was collected (Approved by the Ethics Committee of Zhongshan Hospital, Fudan University, China, NO. B2022‐048R). (iii) FDUZS Cohort 3: 14 cases of HCC who underwent stereotactic body radiation therapy (SBRT) in conjunction with immunotherapy at Fudan University Affiliated Zhongshan Hospital. (iv) TCGA‐LIHC cohort: RNA sequencing (RNA‐seq) and clinical data of 349 HCC samples were obtained from The Cancer Genome Atlas (https://portal.gdc.cancer.gov/).^[^ ^46^ ^]^ (v) ICGC‐LIRI‐JP cohort: RNA‐seq and clinical data from 243 HCC samples were obtained from the International Cancer Genome Consortium (https://dcc.icgc.org/).^[^ ^47^ ^]^ (vi) GSE14520 cohort: Microarray data and clinical information for 225 HCC samples were obtained from the Gene Expression Omnibus database (GEO, https://www.ncbi.nlm.nih.gov/geo/).

### Single‐Cell RNA Sequencing

All fresh samples were processed as follows: For scRNA‐seq, tumor tissue samples were extracted with blades, single cell suspensions were acquired using a Tumor Dissociation Kit (130‐095‐929, Miltenyi Biotec) and a DNaseI (DN25‐100MG, Sigma Aldrich, St. Louis, MO, USA) digestion in a medium (RPMI1640 with 5% fetal bovine serum (FBS)) for 30 min at 37 °C. To remove cell aggregates or other large residual particles from the single‐cell suspension, the cell suspension was filtered through a 40‐um nylon mesh. Red Blood Cell Lysis Solution (10 × ) (Sigma‐Aldrich) and a Dead Cell Removal Kit (Miltenyi Biotec) were used to remove erythrocytes and dead cells, respectively. Library preparation was conducted using a 10 × chromium single‐cell kit following the manufacturer's protocol. Libraries were sequenced using the NovaSeq sequencing platform. The Cell Ranger software pipeline (version 6.0.1) was used to process 10 × raw genomic data. Cell Ranger was applied to demultiplex raw base call files into FASTQ files and for alignment, filtering, barcode counting, and unique molecular identifier (UMI) counting.

### ScRNA‐Seq Data Analysis

Analyses were conducted using Seurat v4 software.^[^ ^48^ ^]^ Combined with the single‐cell gene expression data of all patients, cells with 300–8000 genes detected, 500–100 000 UMI counted, < 30% mitochondrial readings were screened. After filtering, SCTransform with 3000 variable features was used to stabilize the variance of the UMI count.^[^ ^49^ ^]^ The top 20 principal components were used to construct the SNN graph and the Uniform Manifold Approximation and Projection (UMAP) embedding. The “FindClusters” function was adopted for cell clustering analysis. After removing the isolated and unidentified cell subsets, a cluster analysis was performed. The main cell types were identified based on canonical cell‐type markers scored across clusters. The epithelial cell clusters were identified using EPCAM, SOX4, KRT19 or MDK. Glycoproteins JCHAIN, PTPRC, CD68, CD3E or CD79A were used to further identify immune cell clusters. Stromal cell clusters were identified using PECAM1, VWF, ACTA2 or MYL9. Copynumber Karyotyping of Tumors (CopyKAT) v1.0.8 was used to identify malignant epithelial cells.^[^ ^50^ ^]^ Principal component analysis (PCA), SNN map construction, and UMAP embedding were used to determine the optimal subgroup classification for clusters of epithelial cells. For immune cells, sub‐cell types were identified based on cross‐cluster typical cell type labels: B cells were labeled with CD19, CD79A, and MS4A1; CD4, CD3D, CD3E, and TRAC were used to identify CD4+ T cells; CD8A, CD8B, and GZMK were used to identify CD8 + T cells; natural killer cells (NKs) were labeled with GNLY and NKG7; dendritic cells (DCs) were labeled with C1orf54, LGALS2, CD1C, and XCR1; and macrophages were labeled with CD68, FCGR1A, and ITGAX. The differentially expressed genes and DDR markers in each sub‐cluster were computed using the FindMarkers function. Cell cycle phases were scored as described in “Seurat v4”.^[^ ^48^ ^]^ A total of 276 DDR genes were obtained from a previous study^[^ ^51^ ^]^ and the DDR score of each epithelial cell was calculated. Expression of gene sets of “c2.cp.kegg.v7.5.1.symbols.gmt” and “c2.cp.reactome.v2023.1.Hs.symbols.gmt” collection at the Broad Institute.^[^ ^52^ ^]^ Pathway enrichment scores for each immune cell was calculated. The enrichment analysis was performed using the “AUCell” package in R software.^[^ ^53^ ^]^

### Analysis of TME

Single‐sample gene set enrichment analysis (ssGSEA) was used to quantify the level of immune cell infiltration in the TME based on the TCGA‐LIHC and ICGC‐LIRI‐JP cohorts. Genetic characteristics of the immune cells were obtained from Gabriela et al..^[^ ^54^ ^]^ Spearman's analysis was used to calculate the correlation between RECQL4 expression and immune cell infiltration and circulation.^[^ ^55^ ^]^

### Cell Culture

Human HCC cell lines, MHCC97H and HCCLM3, were obtained from the Cell Bank of the Chinese Academy of Sciences, and both were cultured in H‐DMEM containing 10% FBS and penicillin/streptomycin. The mouse HCC cell line, H22, was obtained from the Cell Bank of the Chinese Academy of Sciences and cultured in RPMI 1640 containing 10% FBS and penicillin/streptomycin.

To construct stably overexpressed RECQL4 HCC cells, pcDNA3.1‐RECQL4 was transfected into MHCC97H cells. The cells were then treated with puromycin to select LINC00467 stably overexpressed cells. For the construction of stably silenced RECQL4 HCC cells, sh‐RECQL4‐1, sh‐RECQL4‐2, and sh‐LINC00467‐3 were transfected into HCCLM3 cells. The cells were then treated with puromycin to select stably silenced RECQL4 cells.

### Animals and In Vivo Bioluminescence Assays

C57BL/6 and NSG mice (6–8 weeks old) were purchased from Cyagen Biosciences. cGAS‐deficient mice (Mb21d1^−/−^) were obtained from The Jackson Laboratory (Bar Harbor, ME, USA). STING‐deficient mice (Tmem173gt) were received fromkindly provided by Dr. Liufu Deng's Lab at Shanghai Jiaotong University. Sting‐cKO (Sting^f/f^; ITGAX‐cre) were kindly provided by Dr. Xiaojun Xia's Lab at Sun Yat‐sen University Cancer Center. The mice were maintained under controlled conditions (24 ± 2 °C, 40–70% relative humidity, 12‐h light/12‐h dark cycle) and given a normal laboratory diet and water ad libitum. All mice received humane care in compliance with the institutional animal care guidelines, and the protocols were approved by the local institutional committee. All protocols were conducted in accordance with the Guide for the Care and Use of Laboratory Animals at Zhongshan Hospital, Fudan University. All efforts were made to minimize the number of mice used and their suffering.

For the subcutaneous tumor model, H22 cells (1 × 10^6^) were injected subcutaneously into the mice. Mice carrying Luciferin‐expressing H22 tumors were imaged using an IVIS Lumina K III Imaging System (PerkinElmer, Boston, MA, USA). The mice were administered 150 mg kg^−1^ luciferin (PerkinElmer, Waltham, MA, USA) 5 min before isoflurane anesthesia. The images were processed using Living Image software. The tumor length and width were measured with a caliper, and the volume was calculated as follows: tumor volume = (0.5×length) × (width^2^). When tumor volumes reached 80–100 mm^3^, the mice were exposed to 8 Gy of radiation for three consecutive days.

### RNA Extraction and Quantitative Real‐Time PCR (qRT‐PCR)

Total RNA was extracted using the TRIzol reagent (Invitrogen). Total RNA (1000 ng) was reverse‐transcribed into cDNA according to the manufacturer's instructions (Vazyme). Quantitative PCR (qPCR) was performed using Hieff UNICON® Power qPCR SYBR Green Master Mix (Yeasen) at a final concentration of 0.3 µm, in a sample volume of 10 µL. Data were normalized by the level of beta actin. The 2‐ΔΔCt method was used to calculate the relative expression changes. The primer sequences used for the investigated mouse genes were as follows: actin, forward: GAPDH (Forward – 5′‐GGTGAAGGTCGGTGTGAACG‐3′ and Reverse‐ 5′‐ CTCGCTCCTGGAAGATGGTG‐3′), IFNG (Forward – 5′‐ ATGAACGCTACACACTGCATC‐3′ and Reverse‐ 5′‐ CCATCCTTTTGCCAGTTCCTC‐3′), IFNB1 (Forward – 5′‐ CGTGGGAGATGTCCTCAACT‐3′ and Reverse‐ 5′‐ CCTGAAGATCTCTGCTCGGAC‐3′), CD80 (Forward – 5′‐ GCAGGATACACCACTCCTCAA‐3′ and Reverse‐ 5′‐ AAAGACGAATCAGCAGCACAA‐3′), CD86 (Forward – 5′‐ TCAATGGGACTGCATATCTGCC‐3′ and Reverse‐ 5′‐ CAGCTCACTCAGGCTTATGTTTT‐3′), CXCL10 (Forward – 5′‐ CCAAGTGCTGCCGTCATTTTC‐3′ and Reverse‐ 5′‐ GGCTCGCAGGGATGATTTCAA‐3′).

### Comet Assay

A comet assay was performed according to the manufacturer's instructions (Trevigen). Briefly, cells were trypsinized, washed once with PBS, and resuspended in PBS at a final concentration of 2‐9 × 10^5^ cells per mL. The cell suspension was then combined with pre‐warmed low‐melting agarose poured onto slides. Lysis was performed at 4 °C. Electrophoresis was performed in electrophoresis buffer at 25 V. After DNA precipitation and washing in 70% ethanol, the slides were dried and the DNA was stained with SYBR Gold (Thermo‐Fisher) before epifluorescence microscopy analysis (Olympus Biosystems).

### Clonogenic Assay

Single‐cell suspensions were inoculated into 6‐well plates (1000–8000 cells per well) and treated with IR (0–6 Gy) until cell adherence. After colony formation (approximately 10–14 days), the plates were rinsed with PBS, fixed with methanol, and stained with crystal violet. Colonies containing > 50 cells were counted. Cell survival curves were fitted according to the linear‐quadratic (LQ) formula: surviving fraction: (SF) = exp (−αD − βD^2^).

### Flow Cytometry Analysis

The tumors were harvested and dissociated into single‐cell suspensions. The cells were then blocked with anti‐FcR (clone 2.4G2, BD Pharmingen) and labeled with indicated surface markers for 30 min at 4 °C. For IFN‐γ staining, single‐cells were cultured in the presence of a cell activation cocktail (with Brefeldin A; BioLegend) for 5 h. Cells were permeabilized and stained with intracellular antibodies for 30 min at 4 °C as instructed by the manufacturer. Dead cells were excluded using a LIVE/DEAD Fixable Dead Cell Stain Kit (Invitrogen). The antibodies used in the flow cytometry analysis were anti‐CD45 (clone 30‐F11, Invitrogen), anti‐CD3 (clone 145‐2C11, BioLegend), anti‐CD4 (clone RM4‐5, BioLegend), anti‐CD8 (clone 53–6.7, BioLegend), anti‐IFN‐γ (clone XMG1.2, BioLegend), anti‐CD11c (clone N418, BioLegend), anti‐CD80 (clone 16‐10A1, BioLegend), and anti‐CD86 (clone GL1, Invitrogen). Because the specific reaction with the ovalbumin‐derived peptide SIINFEKL bound to H‐2Kb of MHC class I, anti–H‐2 kb bound to SIINFEKL (clone 25‐D1.16, BioLegend) was used to recognize the tumor‐specific immune cells. Flow cytometry was performed on a FACS Aria III platform (BD Biosciences, San Jose, CA, USA), and the results were analyzed using FlowJo software version 10.4 (TreeStar).

### Preparation of Bone Marrow‐Derived Dendritic Cells (BMDCs)

Bone marrow cells were flushed from the mouse femur and added to red blood cell lysis buffer. The cells were washed and cultured in RPMI‐1640 medium containing 10% FBS supplemented with 20 ng mL^−1^ mGM‐CSF and 20 ng mL^−1^ mIL‐4. Fresh media with supplements were replaced every 2 days. On day 7, the cells were stimulated with the irradiated tumor supernatant overnight according to the grouping.

### IFN‐γ ELISPOT

CD11c^+^ dendritic cells were sorted using a mouse CD11c positive selection kit (BioLegend). After grinding and lysing red blood cells in the spleen, CD8^+^ T cells were sorted using a mouse CD8^+^ naïve T cell isolation kit (BioLegend) according to the manufacturer's instructions. CD11c^+^ cells isolated from BMDC were co‐cultured with purified naïve CD8^+^ T cells at a ratio of 1:10. Spots of IFN‐γ were detected by mouse IFN‐γ precoated ELISPOT kit, according to the manufacturer's instructions (DaYou, 2 210 005). Spots were recognized by an automated ELISPOT reader (Mabtech IRIS FluoroSpot/ELISpot) using the RAWspot technology for multiplexing at the single‐cell level.

### Immunofluorescence and Immunohistochemistry

For immunofluorescence, cells were fixed in 4% paraformaldehyde (PFA) in PBS for 20 min at room temperature (RT), washed twice with PBS, and permeabilized with 0.5% Triton X‐100 in PBS for 10 min. After two additional washes, cells were blocked with 2% bovine serum albumin (BSA) and 2% FBS in PBS (IFF) for 1 h at RT. The cells were then incubated with antibodies in IFF at 4 °C overnight. They were then washed three times with PBS, each for 10 min, followed by incubation with FITC‐conjugated secondary antibodies and 1 µg/ml of DAPI in IFF for 1 h at RT. The cells were then washed three times with PBS and the slides were examined using fluorescence microscopy.

For immunohistochemistry (IHC), fresh tumor tissues were fixed with 4% paraformaldehyde and embedded in paraffin, then cut into 5 µm sections. After dewaxing, rehydration, antigen repair, and blocking, the slides were incubated with primary antibodies overnight. On day 2, the slides were incubated with horseradish peroxidase‐labelled or fluorescent‐conjugated secondary antibodies. DAB was used to visualize the reaction, and hematoxylin was used to label the nuclei.

### Statistical Analysis

The sample sizes were determined based on pilot experiments and previous studies conducted in our laboratory. Two groups were compared using Welch's t‐test or the Mann–Whitney U test (both two‐sided), as indicated, after testing for normal distribution using the Shapiro–Wilk test. For the correlation analysis, we calculated the Spearman correlation coefficient. All statistical analyses were performed using GraphPad Prism 7 (GraphPad Software, San Diego, CA, USA) and R software (version 4.2.0). Statistical significance was set at *P* < 0.05.

### Ethics Approval Statement

This study was approved by the Institutional Review Board of the Zhongshan Hospital, Fudan University (B2022‐048R) and Guangdong Provincial People's Hospital (KY‐Q‐2022‐379‐01). The study was performed in accordance with the Helsinki Declaration and Rules of Good Clinical Practice. All participants signed written informed consents after fully explained.

## Conflict of Interest

The authors declare no conflict of interest.

## Authors Contributions

W.H., Y.Z., and S.W. contributed equally to this work. S.S.D., Q.G., and M.Y. designed the study. W.F.H., Y.Z., S.W.W., and Z.J.L. performed acquisition of data and interpretation of data. W.F.H. performed drafting of the manuscript. All authors interpreted the data, contributed to critical revision of the manuscript, administrative, technical, or material support. All authors read and approved the final manuscript.

## Supporting information

Supporting Information

## Data Availability

The method section describes all the data utilized for analysis and processing in this study. On reasonable request, the corresponding author will provide the data and code supporting the conclusions of this work.
