# Supplementary material for: RECQL4 Inhibits Radiation‐Induced Tumor Immune Awakening via Suppressing the cGAS‐STING Pathway in Hepatocellular Carcinoma
Source: Adv Sci (Weinh). 2024 Feb 21;11(16):2308009. doi: 10.1002/advs.202308009 (PMC11040365; doi:10.1002/advs.202308009)

ADVANCED SCIENCE
Open Access

Supporting Information

for *Adv. Sci.*, DOI 10.1002/advs.202308009

RECQL4 Inhibits Radiation-Induced Tumor Immune Awakening via Suppressing the cGAS-STING Pathway in Hepatocellular Carcinoma

*Weifeng Hong, Yang Zhang, Siwei Wang, Zongjuan Li, Danxue Zheng, Shujung Hsu, Jian Zhou, Jia Fan, Zhesheng Chen, Xiaojun Xia, Zhaochong Zeng, Qiang Gao**, *Min Yu** *and Shisuo Du**

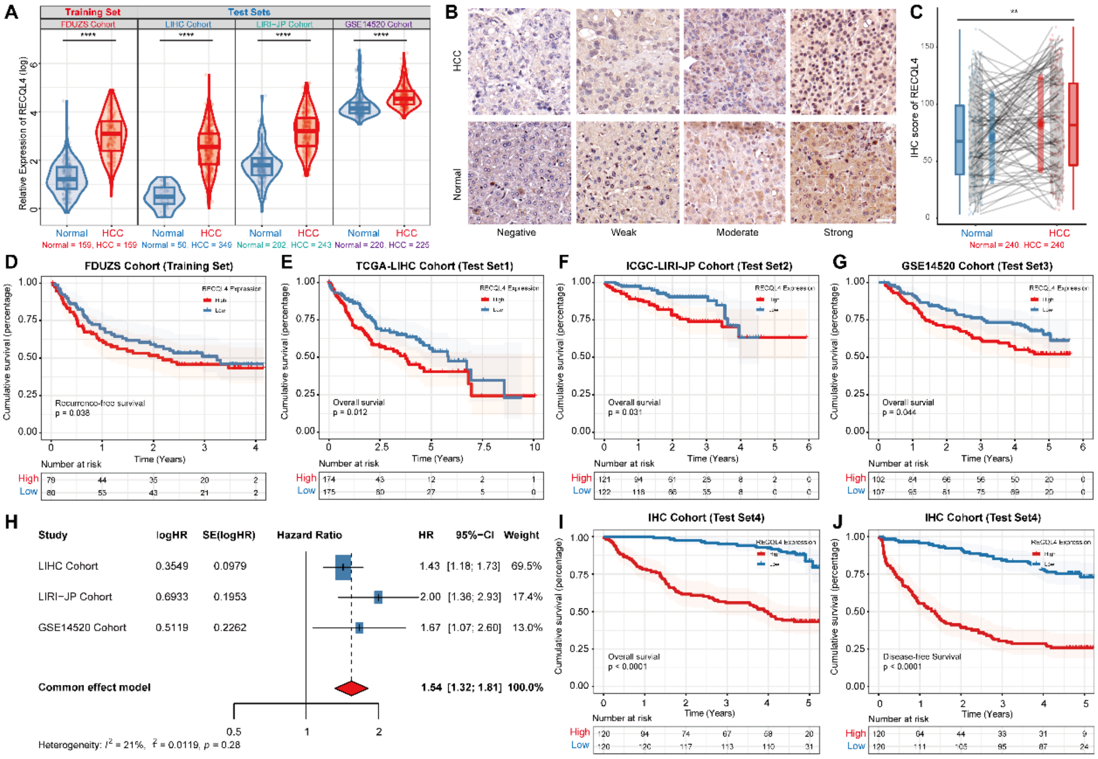

Figure S1. (A). Pie chart showing cell ratio of malignant and non-malignant cells detected by CopyKAT. (B–N). Density plot showing the expression distribution of 13 intersected genes in epithelial cells.

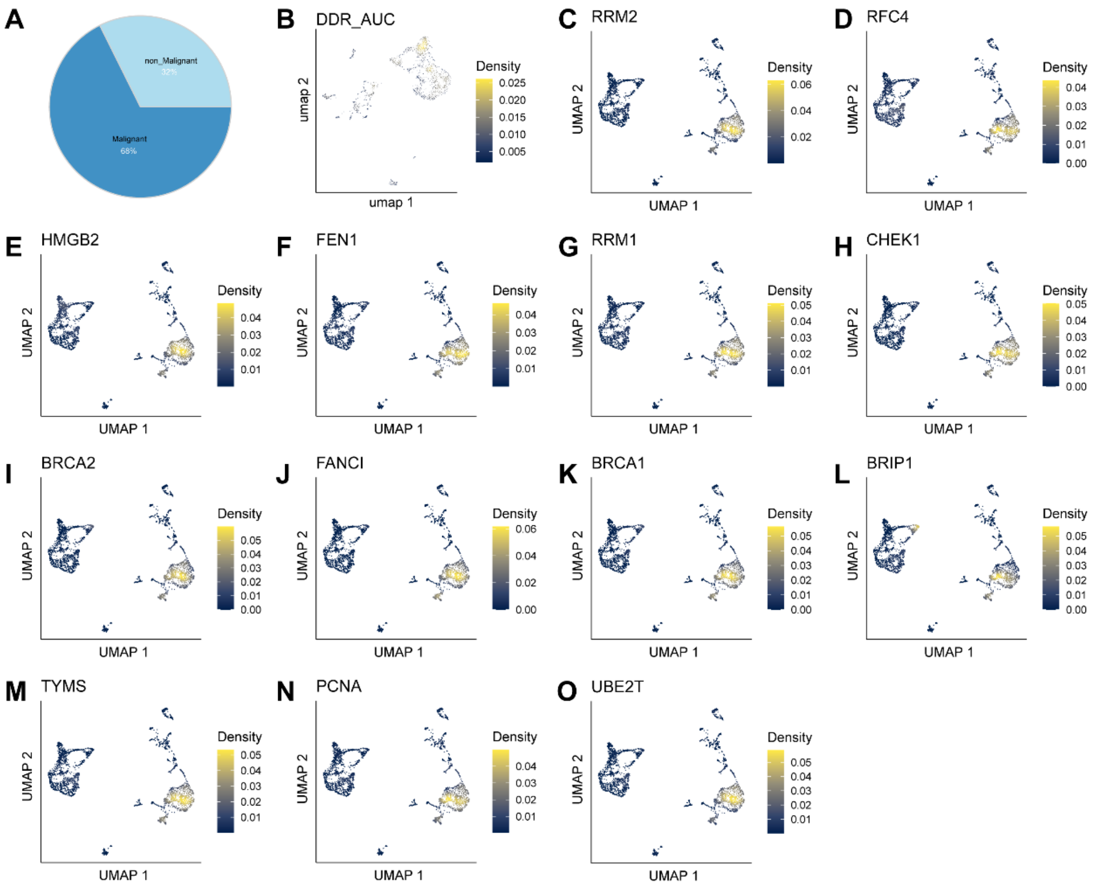

Figure S2. (A and B) qRT-PCR and Western blotting analyses show the relative expression of RECQL4 in eight cell lines. (C and D) qRT-PCR analysis shows the efficiency of stable transfection (overexpression and knockdown) of RECQL4.

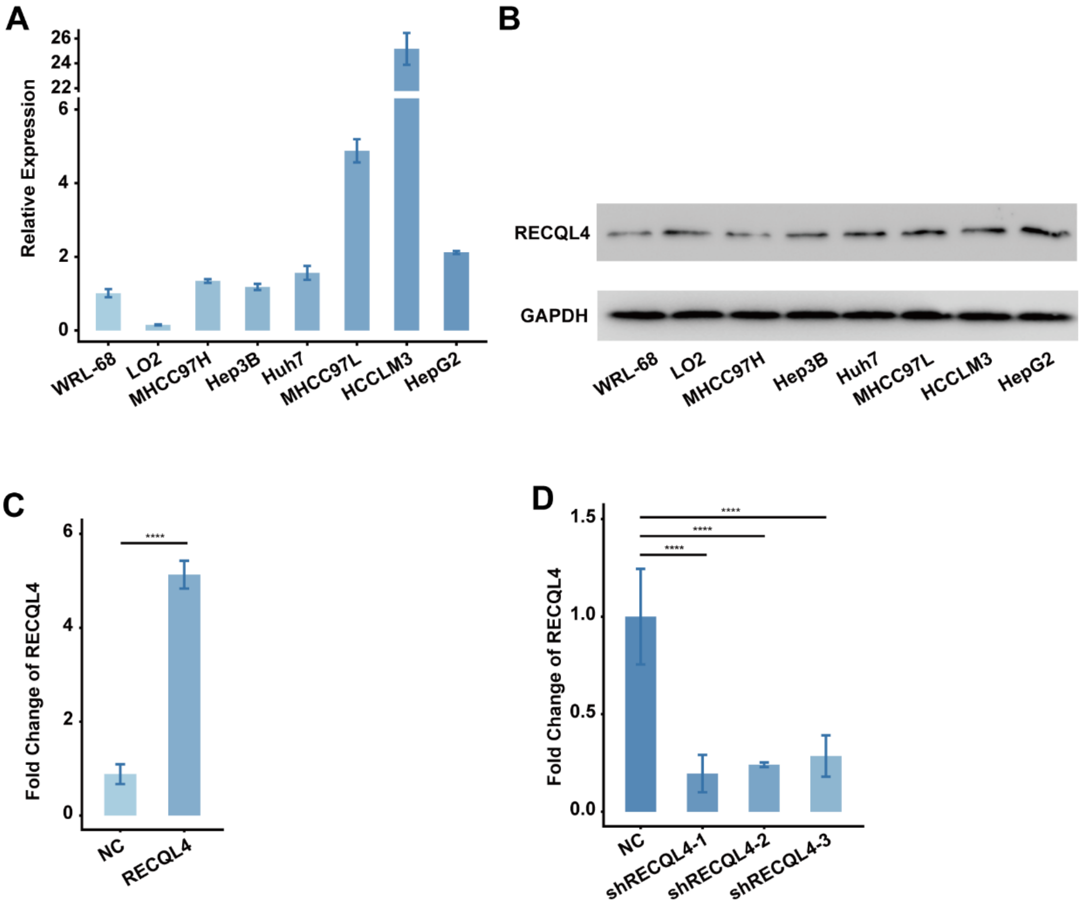

Figure S3. (A) Workflow of subcutaneous HCC transplantation RT model in mice. HCC cells were injected subcutaneously, and when the tumor volume reached ~100 mm3, mice received 8 Gy radiation for three consecutive days. (B) Workflow of subcutaneous HCC transplantation RT model in mice. (C) Bioluminescence images of C57BL/6 mouse HCC tumors.

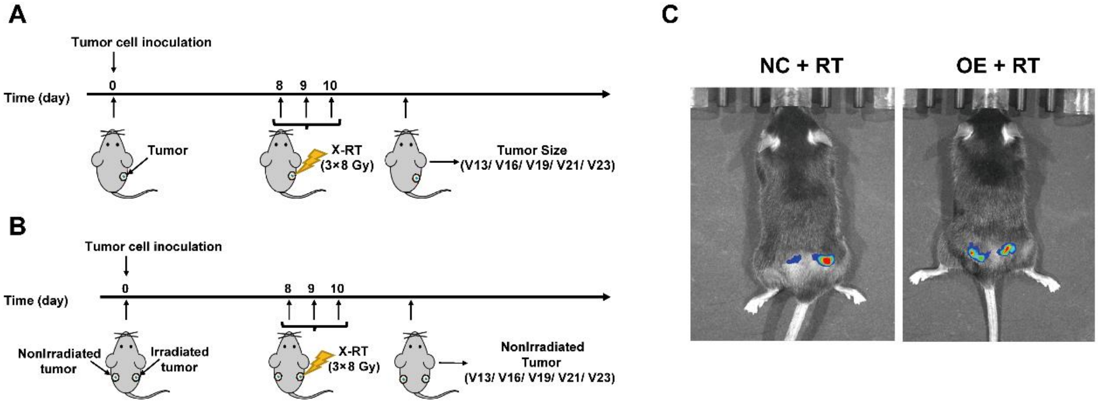

Figure S4. The expression distribution of marker genes in immune cells.

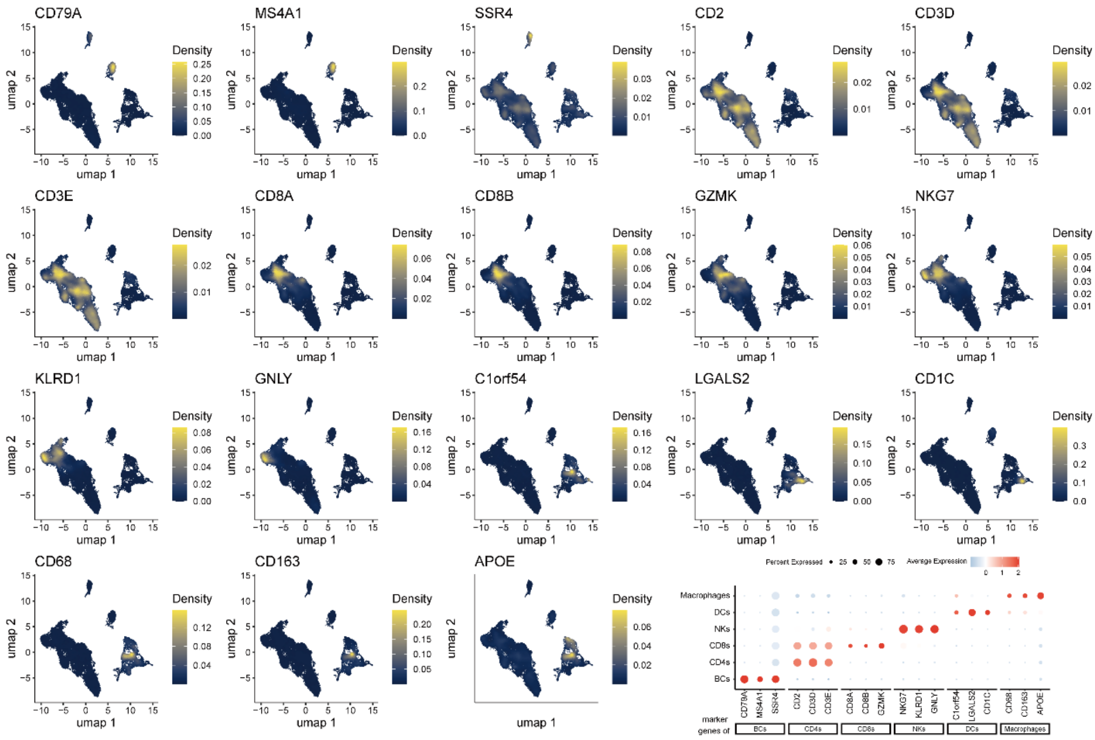

Figure S5. (A) Flow cytometric analysis of tumor-infiltrating CD3+ T cell population (Live/CD45+/CD3+ cells), CD8+ T cell population (Live/CD45/CD3/CD8/CD69), and antigen-specific dendritic cells (CD45+/CD11c+/SIINFEKL+ cells/CD80) in STING-deficient mice tumor tissue and draining lymph nodes. (B) Representative image of ELISPOT assay detecting IFN-γ secretion in STING-mutation mice. (C) Flow cytometric analysis of tumor-infiltrating CD3+ T cell population, CD8+ T cell population, and antigen-specific dendritic cells in cGAS-deficient mice tumor tissue and draining lymph nodes. (D) Representative image of ELISPOT assay detecting IFN-γ secretion in cGAS-KO mice.

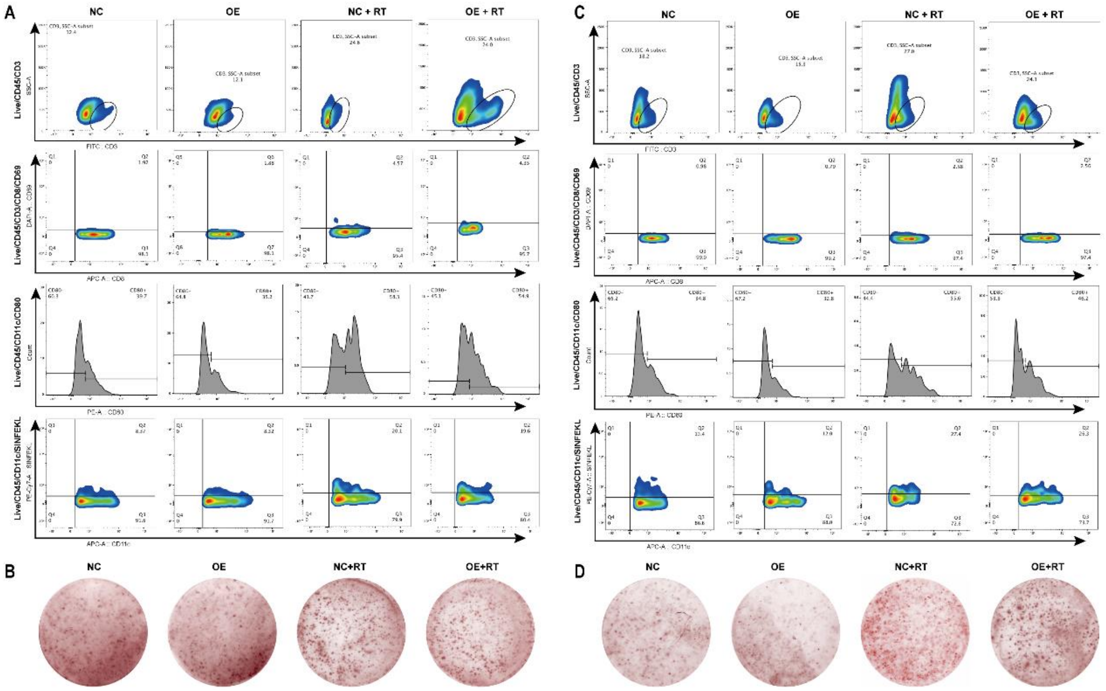

Figure S6. (A–D) qRT-PCR quantification of CD80 (A), CD86 (B), CXCL10 (C), and IFN-β (D) expression in tumor-draining lymph node tissue. (E and F) Purified CD11c+ DCs were co-cultured with initial CD8+ T cells, and IFN-γ secretion was detected by ELISPOT assay. The representative data shown are from six mice per group. Data are presented as mean ± SEM.

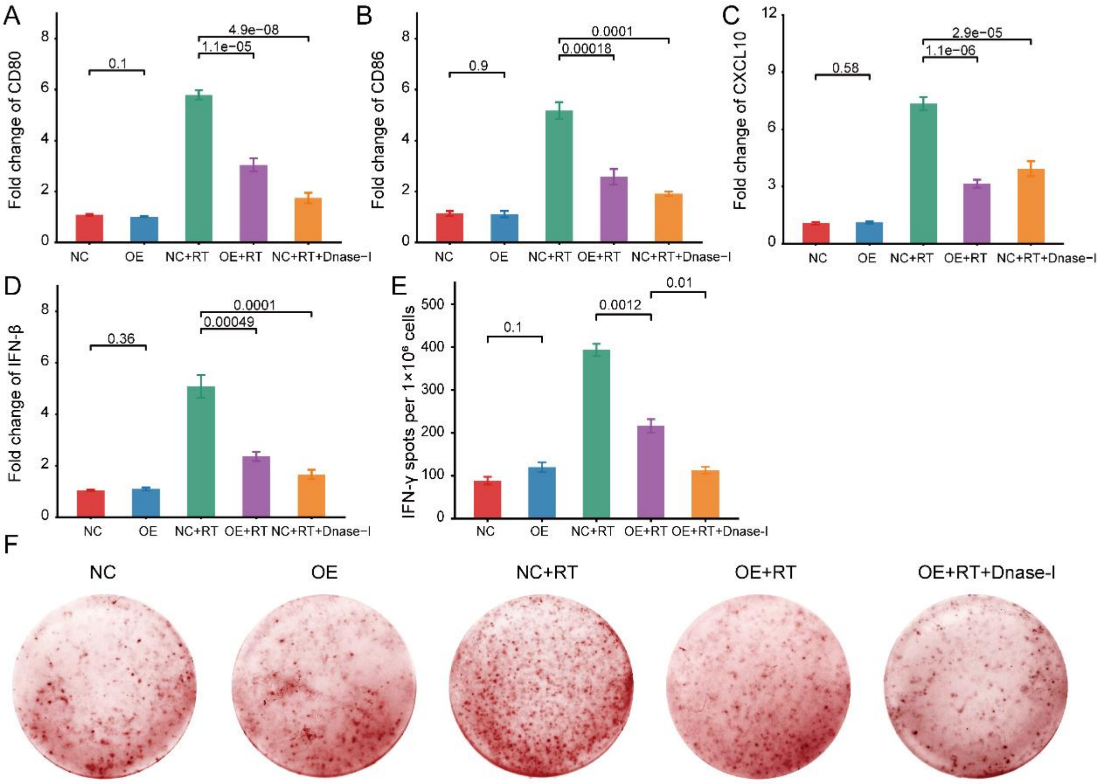

Figure S7. Representative images and statistical analysis of RECQL4 IHC staining intensity in HCC to the combined therapy of SBRT and immunotherapy (left panel). Scale bar, 50 μm. Proportional graph of responsiveness by high or low RECQL4 expression (right panel). CR, complete response; PR, partial response; SD, stable disease; PD, progressive disease.

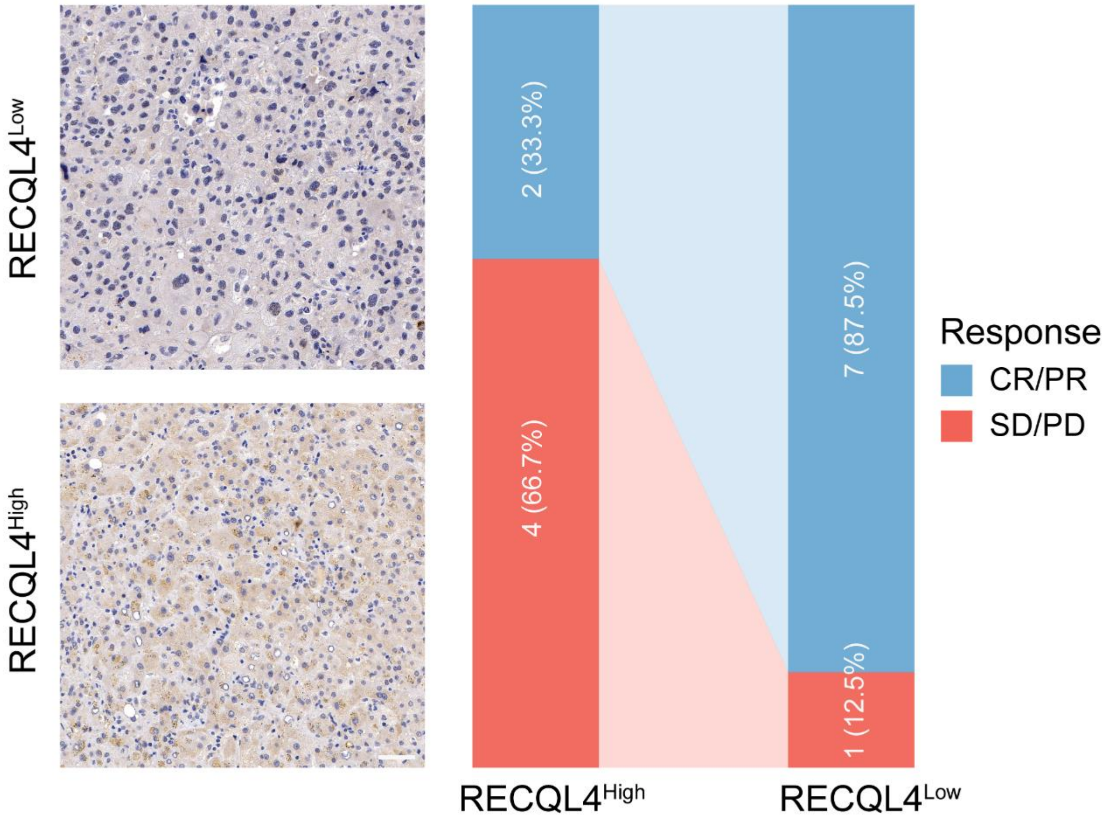

Supplement: Supplementary file 1 — Supporting Information [file ADVS-11-2308009-s001.pdf]
